# Rational design of new cyclic analogues of the antimicrobial lipopeptide tridecaptin A_1_†
†Electronic supplementary information (ESI) available. See DOI: 10.1039/c8cc05790g


**DOI:** 10.1039/c8cc05790g

**Published:** 2018-09-04

**Authors:** Ross D. Ballantine, Yong-Xin Li, Pei-Yuan Qian, Stephen A. Cochrane

**Affiliations:** a School of Chemistry and Chemical Engineering, David Keir Building, Stranmillis Road, Queen's University Belfast , Belfast BT9 5AG , UK . Email: s.cochrane@qub.ac.uk; b Department of Ocean Science and Division of Life Science, Hong Kong University of Science and Technology , Clear Water Bay , Hong Kong , China

## Abstract

Non-ribosomal peptides (NRPs) are a rich source of antibiotic candidates. However, it was recently discovered that resistance to NRPs can be mediated by d-stereoselective peptidases. The tridecaptins, a class of NRPs that selectively target Gram-negative bacteria, are degraded by the d-peptidase TriF. Through analysis of a solution NMR structure of tridecaptin A_1_, we have rationally synthesized new cyclic tridecaptin analogues that retain strong antimicrobial activity and are resistant to TriF.

## Abstract 2

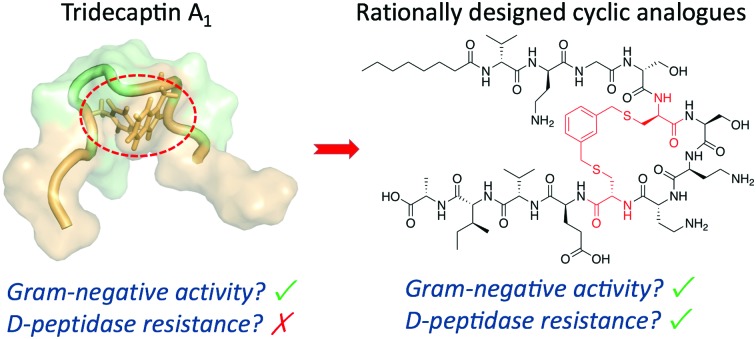
Cyclization of tridecaptin A_1_ imparts stability to the d-peptidase TriF.

## 

Antimicrobial resistance is a major global concern. It has been estimated that if the current rise in infections resulting from multidrug resistant bacteria is not subdued, by 2050 they will cause more deaths than cancer.^1^ Due to the inherent ability of bacteria to develop resistance mechanisms, new antimicrobial compounds and targets will always be needed.^2^ In recent years, there has been a worrying lack of new antibiotics that target Gram-negative bacteria.^3^ These pathogens have an extra layer of protection in the form of the outer-membrane, which precludes the entry of many large-scaffold antibiotics.^4^ Several of the Gram-negative-targeting antibiotics currently undergoing clinical trials are derivatives of known classes (*e.g.* β-lactams/β-lactamase inhibitors) and could therefore be more susceptible to resistance development.^5^ Non-ribosomal peptides (NRPs) are becoming increasingly important in the fight against MDR bacteria, with many new classes being discovered in recent years.^6^d-Amino acid-containing NRPs (dNRPs) are often resistant to peptidases as the vast majority of peptidases only cleave l-peptide bonds. However, recent studies have identified the widespread distribution of d-stereoselective peptidases. For example, the d-peptidase BogQ can degrade the dNRPs bacitracin, rampoplanin and daptomycin, all of which are clinically used antibiotics. Therefore, d-peptidases could pose a major threat to the longevity of NRP antibiotics.^7^

The tridecaptins are a class of linear dNRPs isolated from *Bacillus* and *Paenibacillus* species.^8^ Tridecaptin A_1_ (TriA_1_) is the archetypical member of this class and shows strong activity against Gram-negative bacteria, including multidrug resistant (MDR) strains of *Escherichia coli*, *Klebsiella pneumoniae* and *Acinetobacter baumannii* (Fig. 1A).^9^ TriA_1_ exerts its bactericidal effect by binding to lipid II on the inner-membrane and disrupting the proton-motive force.^10^ The interaction between TriA_1_ and lipid II imbedded in dodecylphosphocholine (DPC) micelles was recently studied by NMR.^10^ In the absence of lipid II, TriA_1_ adopts a tight hairpin-like amphiphilic structure, however a more open looped structure is adopted upon lipid II binding (Fig. 1B). Analysis of this structure suggests that the loop is stabilized by a π-stacking interaction between d-Trp5 and l-Phe9. A previously reported alanine scan of TriA_1_ corroborates the importance of these residues, as substitution of either d-Trp5 or l-Phe9 significantly decreases antimicrobial activity.^11^

**Fig. 1:**
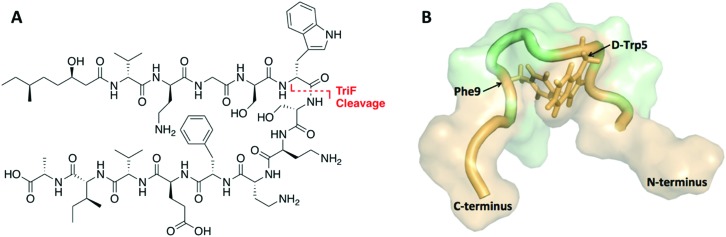
(A) Structure of TriA_1_ with TriF cleavage site shown in red. (B) NMR solution structure of TriA_1_ bound to lipid II (not shown) in DPC micelles (PDB ID: ; 2N5W). A π-stacking interaction that may stabilize the looped structure is shown.

The tridecaptins are attractive antibiotic candidates, owing to their selective activity against Gram-negative bacteria and ease of preparation by solid-phase peptide synthesis (SPPS). However, it was recently discovered that the tridecaptins are hydrolytically cleaved by the d-peptidase TriF at the amide bond between d-Trp5 and Ser6, rendering them inactive.^7^ Although this is likely a self-protection mechanism, similar resistance mechanisms could develop in more pathogenic bacteria, rendering these dNRPs inactive. Macrocyclization is often an effective strategy to improve the stability of peptides towards peptidases.^12^ However, N to C cyclization, which is one of the most commonly used methods to cyclize peptides, is not appropriate for the tridecaptins. Firstly, they are *N*-acylated, making N to C cyclization more difficult. Secondly and most importantly, the lipid II-bound conformation of TriA_1_ places the N- and C-termini far apart as it wraps around lipid II on the cell membrane (Fig. 1B). Given that d-Trp5 and l-Phe9 are in close proximity through a π-stacking interaction, and that d-Trp5 is cleaved by TriF, we rationalized that replacement of this π-stacking interaction with a covalent linkage could impart resistance to TriF, as well as providing a new scaffold of macrocyclic peptides that specifically target Gram-negative bacteria. The generation of cyclic TriA_1_ (cTriA_1_) analogues cyclized between these positions would also corroborate the importance of the looped secondary structure in the mechanism of action of this peptide. Herein, we discuss our synthesis of novel cyclic TriA_1_ analogues that retain strong antimicrobial activity and are resistant to the d-peptidase TriF.

Oct-TriA_1_ (**1**) was first synthesized by Fmoc-SPPS and tested against a model Gram-negative (*Escherichia coli*) and Gram-positive (*Staphylococcus aureus*) indicator strain (Table 1). Replacement of the chiral lipid tail on TriA_1_ with octanoic acid has no effect on antimicrobial activity, therefore N-terminal octanoylation was employed for all analogues.^9^ Consistent with previous reports, Oct-TriA_1_ (**1**) showed potent activity against *E. coli* and weak activity against *S. aureus*. Our initial efforts to prepare cyclic TriA_1_ (cTriA_1_) analogues focused on replacing the d-Trp5-Phe9 π-stacking interaction with an alkene bridge using on-resin ring-closing metathesis (RCM). Derksen and Vederas previously used this strategy to replace a disulfide in the antimicrobial peptide leucocin A.^13^ Oct-TriA_1_-(5-d-Agl, 9-Agl) (**2**) was synthesized by SPPS, however, the full-length peptide proved refractory to on-resin RCM. We rationalized that the peptide might be aggregating, therefore we also attempted the cyclization at the nonapeptide stage of synthesis. However, this also failed. Further attempts involving increased catalyst loading, alternate solvents, increased reaction temperature or chaotropic salts^13^ were also unsuccessful. We postulated that extension of the alkene chains as *S*-allylcysteine (Sac) could facilitate cyclization, however exposure of Oct-TriA_1_-(5-d-Sac, 9-Sac) (**3**) to RCM conditions failed to yield any cyclic product. Neither alkene-containing peptide showed antimicrobial activity <50 μg mL^–1^, suggesting that hydrophobic interactions are not sufficient to stabilize the active conformation of TriA_1_. Previous studies have shown that not all peptides undergo RCM, with yields often highly dependent on peptide sequence.^14^ This situation often arises in peptides containing a large number of hydrophobic amino acids.^15^ Therefore, we believe that the significant hydrophobicity of the C-terminal region of Oct-TriA_1_ causes aggregation on-resin that hinders the RCM reaction. This limitation could be overcome in solution using an aqueous/organic solvent mix, as Oct-TriA_1_ is conformationally flexible in these solvent systems.^11^ However, as the vast excess of chaotropic salts required makes purification difficult,^16^ we directed our efforts towards an alternative cyclization method (Scheme 1).

**Table 1:** Antimicrobial activity of linear TriA_1_ analogues

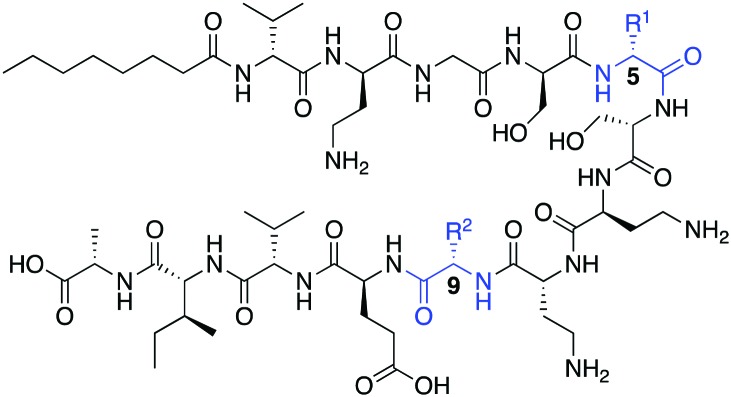				
Peptide	Amino acid		MIC^*a*^	
	**5**	**9**	*E. coli* ^*b*^	*S. aureus* ^*c*^
**1**	d-Trp	Phe	0.39	25
**2**	d-Agl	Agl	>50	>50
**3**	d-Sac	Sac	>50	>50
**4**	d-Cys	Cys	>50	>50

^*a*^MIC = minimum inhibitory concentration. Determined by microbroth dilutions assays and experiments run in duplicate. Values are shown to two significant figures and reported in μg mL^–1^.

^*b*^Strain NCTC 12241.

^*c*^Strain NCTC 10788.

**Scheme 1:**
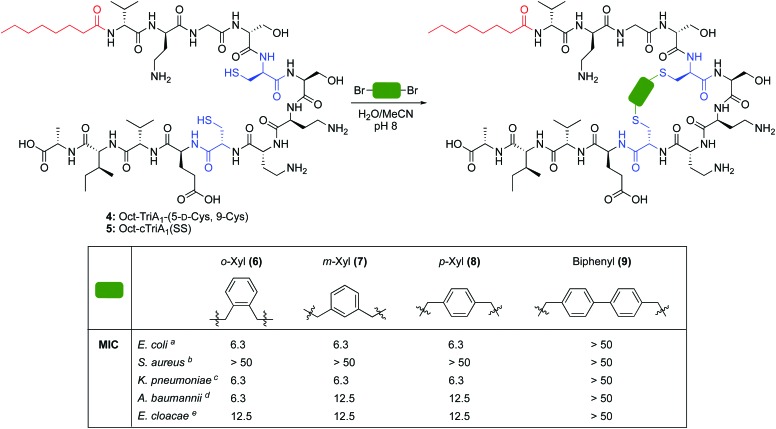
Synthesis of novel cyclic analogues of TriA_1_. MIC = minimum inhibitory concentration. Determined by microbroth dilutions assays and experiments run in duplicate. Values are shown to two significant figures and reported in μg mL^–1^. ^*a*^ Strain NCTC 12241. ^*b*^ Strain NCTC 10788. ^*c*^ Strain NCTC 9633. ^*d*^ Strain NCTC 13304. ^*e*^ Strain NCTC 5920.

The cross-linking of cysteine residues is another method used to staple peptides,^17^ which has been used to prepare cell-penetrant inducers of autophagy^18^ and p53 inhibitors.^19^ In both studies the macrocycle ring size affected activity. Therefore, we directed our efforts towards the generation of cTriA_1_ analogues with varying ring-sizes. Oct-TriA_1_-(5-d-Cys, 9-Cys) (**4**) was first synthesized by SPPS. Next, disulfide **5**, the smallest possible macrocycle, was synthesized from dithiol **4** but showed no antimicrobial activity. Dithiol **4** was then cross-linked with different benzylic cross-linkers to yield Oct-cTriA_1_ analogues **6–9**, which contain increasingly larger macrocyclic rings. Peptides **6–8**, wherein cysteines have been crosslinked with *o*-, *m*- and *p*-Xyl linkers respectively, retained strong activity against *E. coli* (6.3 μg mL^–1^), although none were active against *S. aureus*. Further ring expansion using a biphenyl linker yielded peptide **9**, however this peptide showed no activity at the highest concentrations tested (50 μg mL^–1^). Encouraged by these results, we tested the activity of peptides **6–9** against more clinically significant Gram-negative bacteria, including *Klebsiella pneumoniae*, and the critical tier pathogens *Acinetobacter baumannii* and *Enterobacter cloacae*.^20^ Gratifyingly, peptides **6–8** showed strong activity against all strains. Activity against *A. baumannii* NCTC 13304, which is a carbapenem resistant strain,^21^ is particularly promising as this is currently the WHO's No. 1 priority pathogen.^20^ We next attempted to synthesize cTriA_1_ analogues containing saturated hydrocarbon cross-links. Performing these reactions under the same conditions did not yield any cyclic product, with just linear starting material recovered. Increasing reaction temperatures thermally, or by microwave irradiation, lead to the formation of several side-products, including linear peptides in which one of the cysteine residues had been converted to dehydroalanine. This likely occurs through bisalkylation of a single Cys, followed by elimination of the resulting sulfonium. We therefore moved forward with TriA_1_ analogues **1–9** to study their susceptibility to degradation by the d-peptidase TriF.

The d-stereoselective peptidase TriF is a membrane associated protein found in *Paenibacillus polymyxa* CICC 10580.^7^ TriF_pep_, the soluble periplasmic peptidase domain of TriF, which lacks the signal peptide and four hydrophobic transmembrane helices, was expressed in *E. coli* BL21 cells as a C-terminal His_6_-tag protein construct.^7^ TriA_1_ analogues **1–9** were incubated with TriF_pep_ at 37 °C for 12 h and the reaction mixtures analysed by UPLC-MS. Both Oct-TriA_1_ (**1**) and Oct-TriA_1_(5-d-Sac, 9-Sac) (**3**) were degraded by TriF_pep_ (Fig. 2 and Fig. S1, ESI†), whereas Cys analogue **4** and cTriA_1_ analogues **5–8** were completely resistant (Fig. 2). We were unable to test the stability of Oct-cTriA_1_(biphenyl) (**9**) as it is insoluble in the TriF assay mixture. Although **4** and **5** are resistant to TriF, they also have negligible antimicrobial activity. In contrast, peptides **6–8**, which have been crosslinked with Xyl linkers, retain strong antimicrobial activity and are resistant to TriF. These analogues therefore represent a new scaffold of macrocyclic peptides with selective activity against Gram-negative bacteria. Furthermore, this work highlights the importance of the π-stacking interaction to TriA_1_'s mechanism of action. Improvements in the antimicrobial activity of the cTriA_1_ analogues should be possible through further structure–activity relationship studies.

**Fig. 2:**
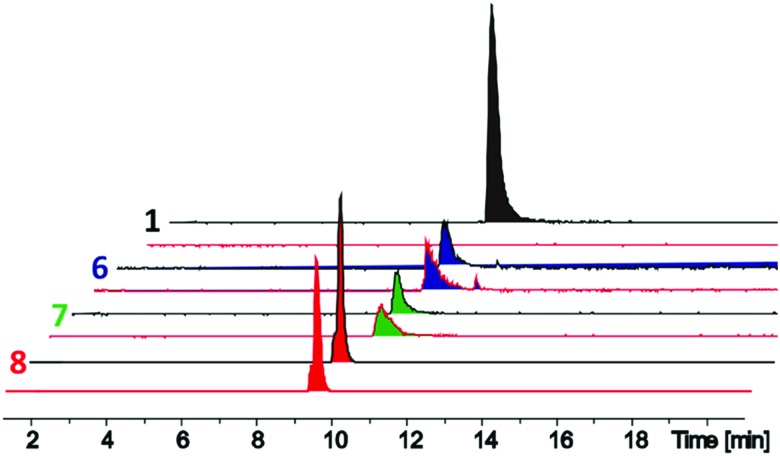
Liquid chromatography-mass spectrometry traces of *in vitro* assays of TriF_pep_ against Oct-TriA_1_ (**1**, black peaks) and *o*-Xyl- (**6**, blue peaks), *m*-Xyl- (**7**, green peaks) and *p*-Xyl- (**8**, red peaks) crosslinked peptides. Standards (black line) without TriF_pep_ and experiments with TriF_pep_ (red line) are shown. Whereas Oct-TriA_1_ is cleaved by TriF_pep_, no corresponding cleavage was observed in TriF_pep_ treated **6–8**.

In conclusion, we have employed a rational design approach to generate new cyclic analogues of tridecaptin A_1_ that are resistant to the d-peptidase TriF. Analysis of an NMR solution structure of TriA_1_ identified a possible cyclization point between positions 5 and 9, which are in close proximity due to a π-stacking interaction. Substitution of d-Trp5 and l-Phe-9 with d-Cys and l-Cys respectively, followed by cross-linking with benzylic di-bromo linkers yielded cyclic TriA_1_ analogues that retain selective activity against Gram-negative bacteria and are resistant to TriF. To the best of our knowledge, the replacement of π-stacking interactions with a covalent linkage to impart peptidase stability is a novel strategy. These structures constitute a new class of Gram-negative-targeting macrocyclic peptides and could be the basis for new antibiotic candidates.

## Conflicts of interest

There are no conflicts to declare.

## Supplementary Material

Supplementary information
