# Supplementary material for: Rational design of new cyclic analogues of the antimicrobial lipopeptide tridecaptin A1
Source: Chem Commun (Camb). 2018 Sep 4;54(75):10634–7. doi: 10.1039/c8cc05790g (PMC6146376; doi:10.1039/c8cc05790g)
Supplement: Supplementary file 1 [file CC-054-C8CC05790G-s001.pdf]

Electronic Supplementary Material (ESI) for ChemComm.
This journal is © The Royal Society of Chemistry 2018

Supplementary Information

FOR

Rational Design of New Cyclic Analogues of the Antimicrobial Lipopeptide Tridecaptin A_1

Ross D. Ballantine,[a] Yong-Xin Li,[b] Pei-Yuan Qian[b] and Stephen A. Cochrane*[a]

[a] School of Chemistry and Chemical Engineering, David Keir Building, Stranmillis Road, Queen's University Belfast, Belfast, UK, BT9 5AG; [b] Department of Ocean Science and Division of Life Science, Hong Kong University of Science and Technology, Clear Water Bay, Hong Kong, China

*To whom correspondence should be addressed. Email: s.cochrane@qub.ac.uk

TABLE OF CONTENTS

Section	Contents	Page Number
I	Materials	S2
II	General procedure for peptide synthesis	S2
III	Purification and analysis of peptides	S2
IV	Unsuccessful attempts to synthesize cyclic TriA_1 analogues by Ring-Closing Metathesis	S3
V	Synthesis of Oct-cTriA_1 (SS) (**5**)	S4
VI	General procedure for peptide cross-linking	S4
VII	Unsuccessful attempts to cross-link peptides using saturated dibromo-linkers	S4
VIII	Antimicrobial testing	S5
IX	Peptide degradation studies with TriF_{pep}	S5
X	TriF_{pep} assays with peptides **2** - **4**	S6
XI	HPLC purification and HRMS analysis of peptides	S7
XII	References	S11

I. Materials

All Proteinogenic Fmoc-amino acids were purchased from CEM. The remaining Fmoc-amino acids, including Fmoc-Agl-OH, Fmoc-D-Agl-OH, Fmoc-D-Cys(Trt)-OH, Fmoc-Dab(Boc)-OH, Fmoc-D-Dab(Boc)-OH, Fmoc-D-*a*Ile-OH, Fmoc-D-Ser(*t*Bu)-OH, Fmoc-D-Trp(Boc)-OH and Fmoc-D-Val-OH were purchased from Fluorochem. Fmoc-Sac-OH and Fmoc-D-Sac-OH were synthesized as previously described.[1,2] [Bis(dimethylamino)methylene]-1*H*-1,2,3-triazolo[4,5-*b*]pyridinium-3-oxidhexafluorophosphate (HATU), 1,2-Bis-(bromomethyl)xylene, 1,3-Bis-(bromomethyl)xylene, 1,4-Bis-(bromomethyl)xylene, 1,2-ethanedithiol (EDT), trifluoroacetic acid (TFA) and triisopropylsilane (TIPS) were purchased from Fluorochem. Ala-2-chlorotrityl resin, 4,4''-Bis(bromomethyl)biphenyl, dichloroethane (DCE), diisopropylethylamine (DIPEA), dimethylsulfoxide (DMSO) and Hoveyda-Grubbs Second Generation Catalyst were purchased from Sigma-Aldrich. Anhydrous lithium chloride was purchased from Alfa-Aesar. HPLC grade acetonitrile (ACN), dichloromethane (DCM), dimethylformamide (DMF) and methanol (MeOH) were purchased from Merck. All chemicals were used without further purification. All materials required for the expression and purification of $TriF_{pep}$, as well as D-peptidase assays have been previously described.[3]

II. General procedure for peptide synthesis

Peptides were synthesized on a CEM Liberty 12 Microwave Peptide Synthesizer. Solid phase synthesis was carried out on a 0.1 mmol scale using Fmoc chemistry on 2-chlorotrityl (2CT) resin preloaded with Ala (0.6 mmol/g). Factory settings were used for all coupling and deprotection cycles. Asymmetrically protected amino acids were used as 0.2 M solutions of DMF with amino acid subunits being coupled using HATU as the activator and DIPEA as the activator base and heated to 70 °C (50 °C for cysteine residues) for 5 minutes. Fmoc residues were deprotected using a 20 % solution of piperidine in DMF. Upon completion of synthesis, the peptide resin was washed with CH_2Cl_2 (3 x 5 mL) in a glass-fritted filter funnel and dried under vacuum suction for 15 min. Dried peptide resin was transferred to a vial containing TFA:TIPS:water (95:2.5:2.5, 10 mL) and heated at 37 °C for 2 h with periodic manual shaking. For cysteine-containing peptides, an alternate cleavage cocktail of TFA:EDT:TIPS:water (95:2.5:2.5:1, 10.1 mL) was used under the same reaction conditions. The resin beads were then removed by filtration through glass wool and the filtrate was concentrated *in vacuo*. Et_2O (5 mL) was added to precipitate crude peptide and the resulting suspension transferred to 2 mL centrifuge tubes. The tubes were centrifuged for 1 min using a microcentrifuge and the liquid decanted. Pellets were resuspended in Et_2O (1 mL), vortexed, centrifuged and the liquid decanted to yield crude peptide pellets.

III. Purification and analysis of peptides

Crude peptide was dissolved in minimal 1:1 0.1% aqueous TFA/ACN and purified by Reversed-Phase High Performance Column Chromatography (RP-HPLC). Purification was performed on a Perkin Elmer HPLC system composed of a 200 series binary pump, UV/Vis detector, vacuum degasser, Rheodyne 7725i injector equipped with a 2 mL sample loop and Phenomenex Luna C18 column (5 µm, 250 x 21.2 mm). The system was operated using ThermoFisher Chromeleon 7.2 software. Runs were performed at a flow rate of 10 mL/min with UV detection at 220 nm. Solvent A = 0.1 % TFA in MilliQ water and solvent B = 0.1 % TFA in ACN. A gradient method was employed, starting from 20 % B and 80 % A for 5 min, ramping up to 55 % B over 30 min, then ramping up to 95 % B over 3 min, remaining at 95 % B for 3 min, ramping down to

20 % B over 2 min before staying at 20 % B for 5 min. Product containing fractions were pooled, concentrated under vacuum to remove ACN, frozen and then lyophilized to yield pure peptides as white flocculent solids. High resolution mass spectrometry (HRMS) spectra of all peptides were recorded by Analytical Services and Environmental Projects (ASEP) at Queen's University Belfast on a Waters LCT Premier ToF mass spectrometer using the electrospray ionisation (ESI) technique.

IV. Unsuccessful attempts to synthesize cyclic TriA$_1$ analogues by Ring-Closing Metathesis

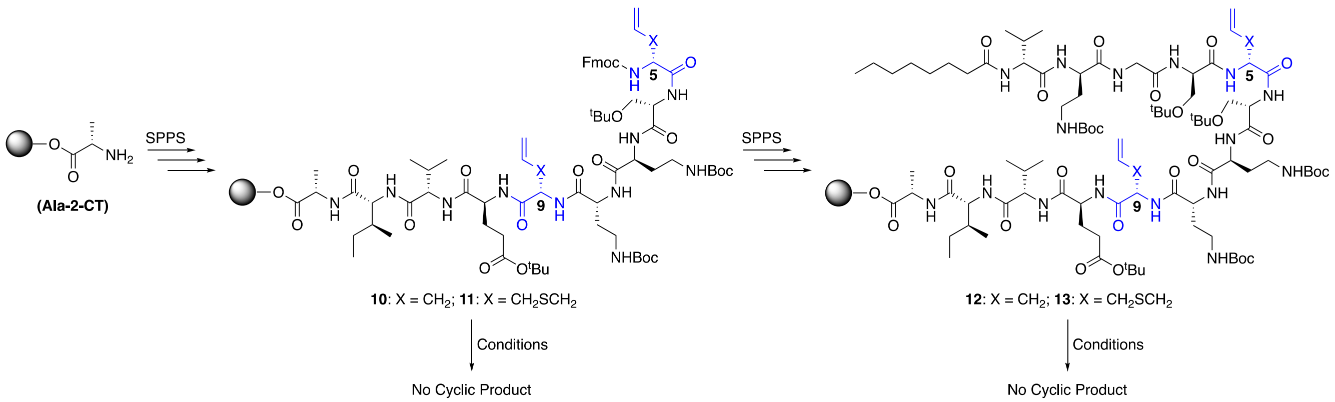

Method 1 – RCM of full-length peptides on resin: Oct-TriA$_1$ (5-D-Agl, 9-Agl)-2CT (**12**) and Oct-TriA$_1$ (5-D-Sac, 9-Sac)-2CT (**13**) were synthesized using Fmoc-SPPS. A small amount of peptide was cleaved from resin (~ 1 mg) and analysed by HRMS as previously described to confirm the correct starting material had been prepared. The dried resin of each synthesis was weighed, and the total mass assumed to correlate to 0.1 mmol peptide. The on-resin peptide (0.0882 mmol) was added to a solution of Hoveyda-Grubbs Second Generation Catalyst (11 mg, 0.0176 mmol, 0.2 equiv.) in anhydrous and degassed DCE (5 mL), a waterless condenser was attached, and the resulting suspension heated at 70 °C under argon for 72 h. The reaction mixture was then cooled to room temperature, DMSO (0.02 mL, 0.281 mmol) added and the suspension gently stirred for a further 24 h. The mixture was filtered through a glass-fritted filter funnel and the resin washed with DCM and MeOH. The peptide was then cleaved from resin and the crude peptide purified by HPLC as described above. No cyclic products were found.

Method 2 – RCM of full-length peptides on resin pre-swollen with lithium chloride: This method was performed according to a previously reported literature procedure.[4] The resin-bound peptide (0.100 mmol) was added to a manual SPPS vessel and bubbled in dry DCM (10 mL) for 30 min with argon. The resin was subsequently washed with degassed DCE (3 x 5 mL), followed by bubbling in a 0.8 M solution of lithium chloride (3 mL) for 2 min. The resin was then filtered and washed with DMF (2 x 5 mL). Bubbling with the lithium chloride solution was repeated twice more before a final wash with DCE (3 mL). The pre-swollen peptide resin was then added to a solution of Hoveyda-Grubbs Second Generation Catalyst (11.3 mg, 0.0180 mmol) in anhydrous and degassed DCE (3 mL), a waterless condenser was attached, and the resulting suspension refluxed under argon for 70 h. The reaction mixture was cooled to ambient temperature and a second batch of catalyst (39.5 mg, 0.0630 mmol) added. The resulting mixture was then refluxed for a further 24 h. The reaction mixture was then cooled to ambient temperature, filtered and the resin washed with DCM (3 x 5 mL) and DMF (3 x 5 mL). The resin was then suspended

in DMF (2 mL) and DMSO (1.92 mL, 27.0 mmol) and bubbled with argon for 12 h. The resin was then washed with DMF (3 x 10 mL), DCM (3 x 5 mL) and MeOH (3 x 5 mL). The peptide was then cleaved from resin and the crude peptide purified by HPLC as described above. No cyclic products were found.

Method 3 – RCM of TriA$_1$ (5-D-Agl, 9-Agl)-2CT nonapeptide *on resin*: Truncated TriA$_1$ (5-D-Agl, 9-Agl)-2CT nonapeptide (**10**) was synthesized using Fmoc-SPPS. RCM of this peptide was then attempted using method 1. No cyclic products were found.

Method 4 – RCM of TriA$_1$ (5-D-Agl, 9-Agl)-2CT nonapeptide *on resin pre-swollen with lithium chloride*: RCM of Truncated TriA$_1$ (5-D-Agl, 9-Agl)-2CT nonapeptide (**10**) was attempted using method 2. No cyclic products were found.

V. Synthesis of Oct-cTriA$_1$ (SS) (5)

Oct-TriA$_1$ (5-D-Cys, 9-Cys) (**4**) was synthesized by Fmoc-SPPS as described above. The crude peptide from a 0.1 mmol synthesis was re-dissolved in 1:1 water/ACN (16 mL) to make a 6.25 mM stock solution. An aliquot of this stock solution (2.0 mL, 0.0125 mmol) was added to a solution of TFA (1.7 mL, 22.2 mmol) in distilled water (32 mL). The pH was adjusted to pH 6 with solid ammonium bicarbonate, followed by the addition of DMSO (6.8 mL, 7.48 mol). The reaction mixture was stirred for 5 h at ambient temperature and diluted with 1:1 0.1 % aqueous TFA/ACN. The resulting solution was concentrated *in vacuo*, re-dissolved in 1:1 0.1 % aqueous TFA/ACN (18 mL) and purified by RP-HPLC.

VI. General procedure for peptide cross-linking

An aliquot of thiol **4** stock solution (6.25 mM, 2.0 mL, 0.0125 mmol) was added to a 20 mM solution of ammonium bicarbonate in 1:1 water/ACN (12.2 mL). The pH was adjusted to pH 8 using a 1 M solution of ammonium bicarbonate. A solution of the appropriate dibromo linker (1.46 equiv, 0.0183 mmol) in ACN (1 mL) was then added and the resulting reaction mixture stirred for 2 hours at ambient temperature. TFA (0.1 mL) was then added and the resulting solution concentrated *in vacuo* to remove ACN. The solution was then frozen, lyophilised and re-dissolved in minimal 1:1 0.1% aqueous TFA/ACN for purification by RP-HPLC.

VII. Unsuccessful attempts to cross-link peptides using saturated dibromo-linkers

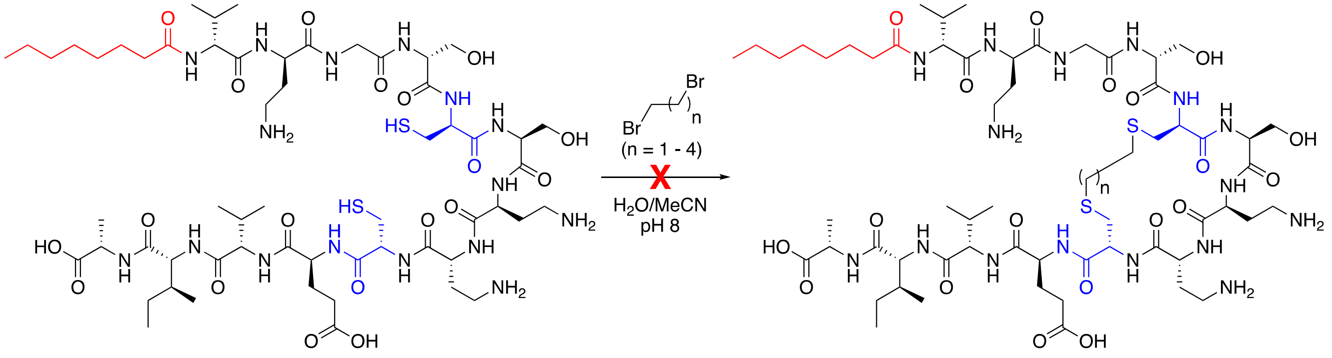

Oct-TriA$_1$-(5-D-Cys, 9-Cys) (**4**)

Method 1 – Cross-linking reactions performed at ambient temperature: Reactions were first performed according to the general procedure for peptide cross-linking, with the reaction time adjusted to 24 h. In all instances only unreacted starting material was observed.

Method 2 – Cross-linking reactions performed at 100 °C: Reactions were performed according to the general procedure for peptide cross-linking, with the reaction time adjusted to 1 h and temperature increased to 100 °C. In these experiments no cyclic products were recovered by HPLC, however peptides in which one of the cysteine residues had been converted to dehydroalanine were identified by HRMS ([M+H]+ calcd for $C_{58}H_{103}N_{16}O_{19}S$ 1359.7306, found 1359.7301).

Method 3 – Cross-linking reactions performed at 100 °C with microwave irradiation: An aliquot of thiol **4** stock solution (1.0 mL, 0.006 mmol) was added a 20 mM solution of ammonium bicarbonate in 1:1 water/ACN (1 mL). The pH was adjusted to pH 8 using a 1 M solution of ammonium bicarbonate. The dibromo linker (1 equiv, 0.006 mmol) was then added directly by pipette. The resulting solution was flushed with argon and microwave irradiated using a CEM Discover at 100 °C for 10 minutes (20 W) with a 2 min ramp. In these experiments no cyclic products were recovered by HPLC, however peptides in which one of the cysteine residues had been converted to dehydroalanine were found as described above.

VIII. Antimicrobial testing

All minimum inhibitory concentrations (MICs) were determined according to Clinical and Standards Laboratory Institute (CLSI) guidelines.[5] Briefly, peptides were dissolved in Muller-Hinton Broth (MHB) and serial dilutions made across a 96 well plate. Each well was inoculated with a suspension of the required bacterial strain to reach a final inoculum of 5 x 10^5 colony forming units per mL. The MIC was taken as the lowest concentration with no visible growth after 18 hours.

IX. Peptide degradation studies with $TriF_{pep}$

$TriF_{pep}$ was expressed and purified as previously described.[3] An assay mixture (total volume 30 µL in 25% phosphate-buffered saline (PBS) buffer, pH 7.4) containing $TriF_{pep}$ (2.0 µM) and the desired peptide (added as a DMSO solution to give a final concentration of 20 µM) was prepared and incubated for 12 h at 37 °C. Three volumes of cold MeOH (90 µL) were added and the resulting mixture incubated at −80 °C for 1 h to precipitate protein. The samples were centrifuged at 15,000 rpm for 10 min and 2 µL of the supernatant was analyzed by UPLC–MS system as previously described.[3]

X. $TriF_{pep}$ assays with peptides 2 – 4

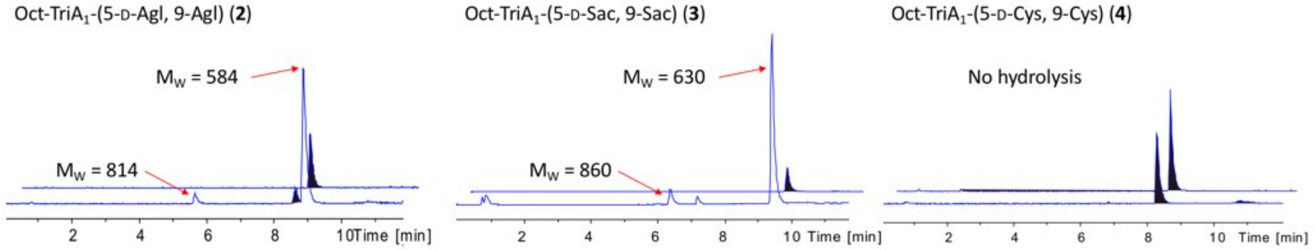

Fig S1 Liquid chromatography-mass spectrometry traces of *in vitro* assays of $TriF_{pep}$ against Oct-$TriA_1$-(5-D-Agl, 9-Agl) (**2**, left), Oct-$TriA_1$-(5-D-Sac, 9-Sac) (**3**, middle) and Oct-$TriA_1$-(5-D-Cys, 9-Cys) (**4**, right). Standards (top line, blue peak) without $TriF_{pep}$ and experiments with $TriF_{pep}$ (bottom line) are shown. The M_w of fragmentation products is shown where appropriate. Peptides **2** and **3** are hydrolyzed by $TriF_{pep}$ but **4** is resistant.

XI. HPLC and HRMS analysis of peptides

Peptide	Name	Chemical Formula	Calcd Exact Mass	Mass found	Calcd	Overall Yield [%]
**1**	Oct-$TriA_1$	$C_{72}H_{113}N_{17}O_{19}$	1519.8399	760.9263 $[M + 2H]^{2+}$	760.9278	11
**2**	Oct-$TriA_1$ (5-D-Agl, 9-Agl)	$C_{62}H_{106}N_{16}O_{19}$	1380.7977	691.4092 $[M + 2H]^{2+}$	691.4068	6
**3**	Oct-$TriA_1$ (5-D-Sac, 9-Sac)	$C_{64}H_{112}N_{16}O_{19}S_2$	1472.7731	737.4164 $[M + 2H]^{2+}$	737.3944	4
**4**	Oct-$TriA_1$ (5-D-Cys, 9-Cys)	$C_{58}H_{104}N_{16}O_{19}S_2$	1392.7105	697.3827 $[M + 2H]^{2+}$	697.3631	18
**5**	Oct-c$TriA_1$ (SS)	$C_{58}H_{102}N_{16}O_{19}S_2$	1390.6949	696.3559 $[M + 2H]^{2+}$	696.3553	6
**6**	Oct-c$TriA_1$ (*o*-Xyl)	$C_{66}H_{110}N_{16}O_{19}S_2$	1494.7575	748.3911 $[M + 2H]^{2+}$	748.3866	14
**7**	Oct-c$TriA_1$ (*m*-Xyl)	$C_{66}H_{110}N_{16}O_{19}S_2$	1494.7575	746.3709 $[M - 2H]^{2-}$	746.3709	50
**8**	Oct-c$TriA_1$ (*p*-Xyl)	$C_{66}H_{110}N_{16}O_{19}S_2$	1494.7575	746.3708 $[M - 2H]^{2-}$	746.3709	23
**9**	Oct-c$TriA_1$ (biphenyl)	$C_{72}H_{114}N_{16}O_{19}S_2$	1570.7888	786.4009 $[M + 2H]^{2+}$	786.4022	5

Table S1. Peptide number, name, chemical formula, exact mass, mass found and overall yield for peptides **1** – **8**.

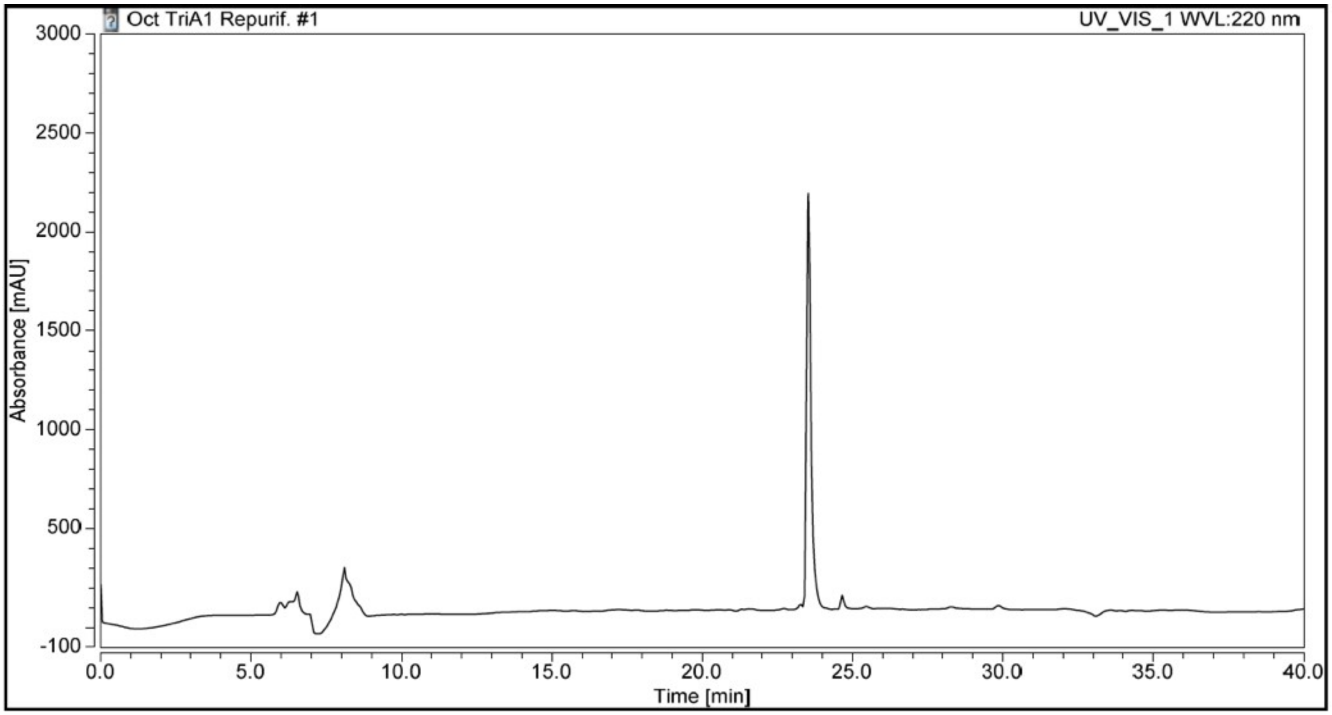

Fig. S2. HPLC trace showing the reinjection of purified Oct-TriA$_1$ (**1**). The peptide eluted as a single peak at 23.5 min using the HPLC method outlined in part III.

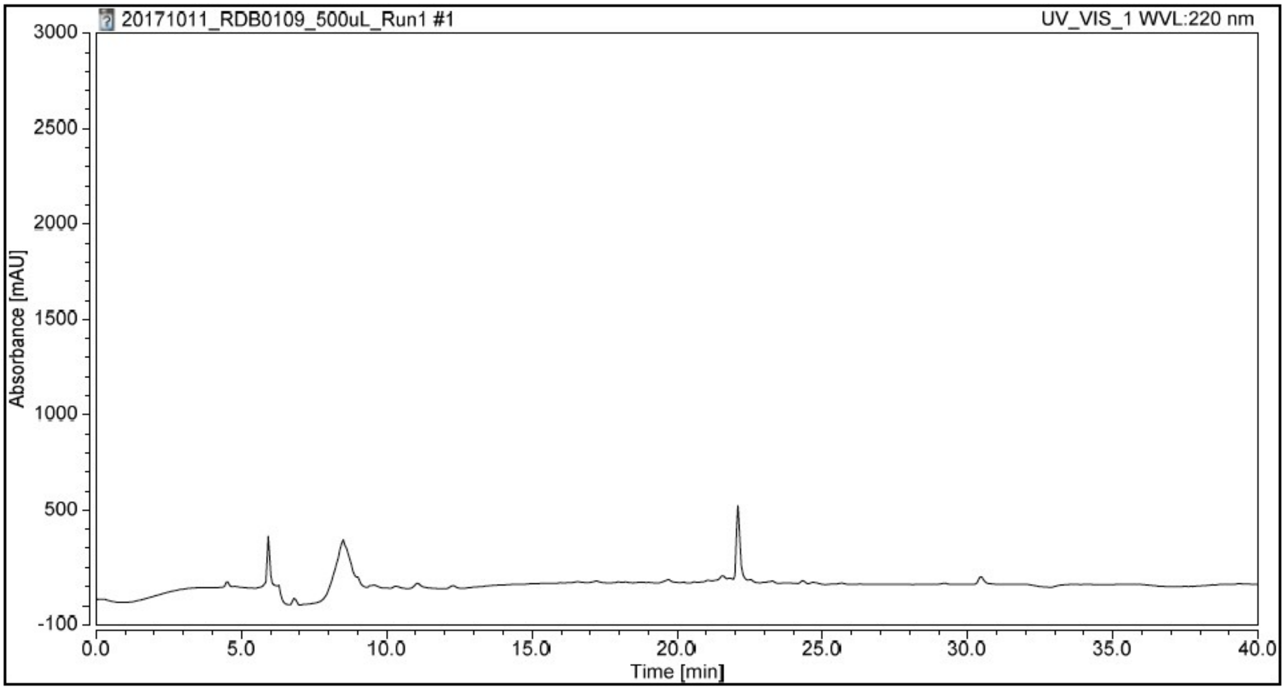

Fig. S3. HPLC trace showing the reinjection of purified Oct-TriA$_1$ (5-D-Agl, 9-Agl) (**2**). The peptide eluted as a single peak at 22.1 min using the HPLC method outlined in part III.

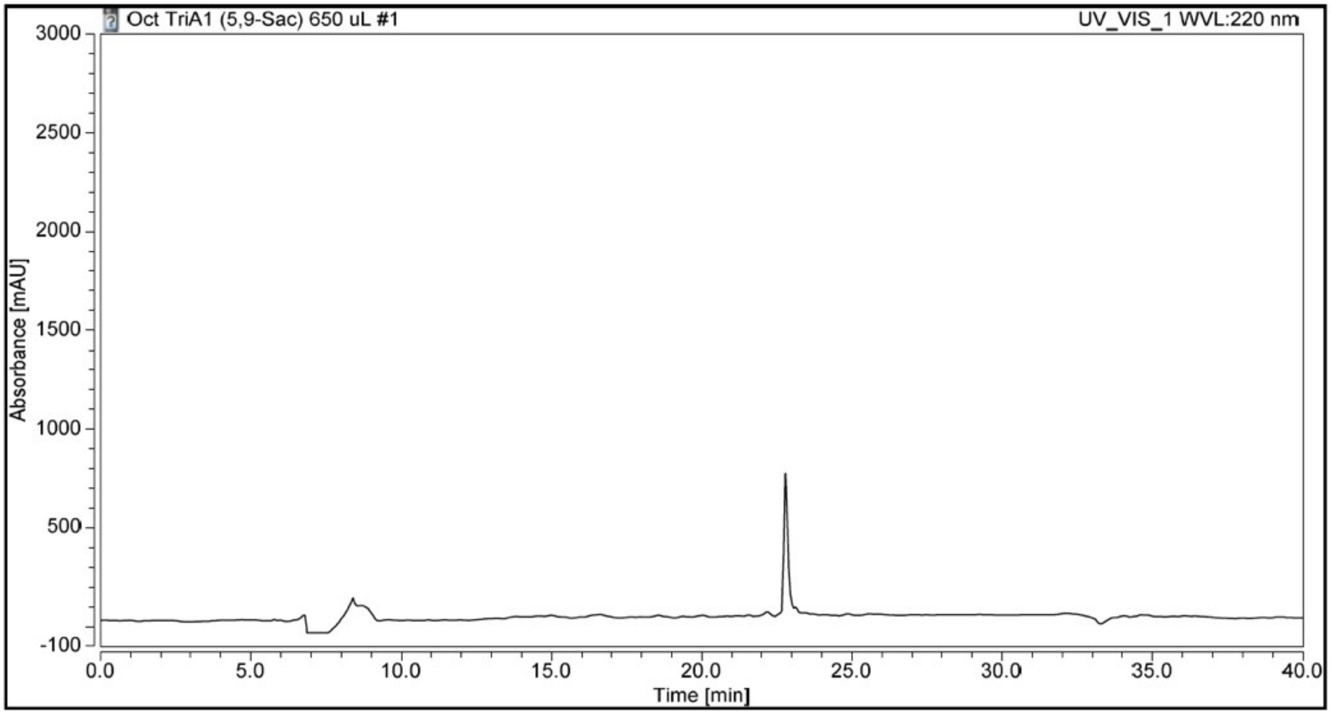

Fig. S4. HPLC trace showing the reinjection of purified Oct-TriA$_1$ (5-D-Sac, 9-Sac) (**3**). The peptide eluted as a single peak at 22.8 min using the HPLC method outlined in part III.

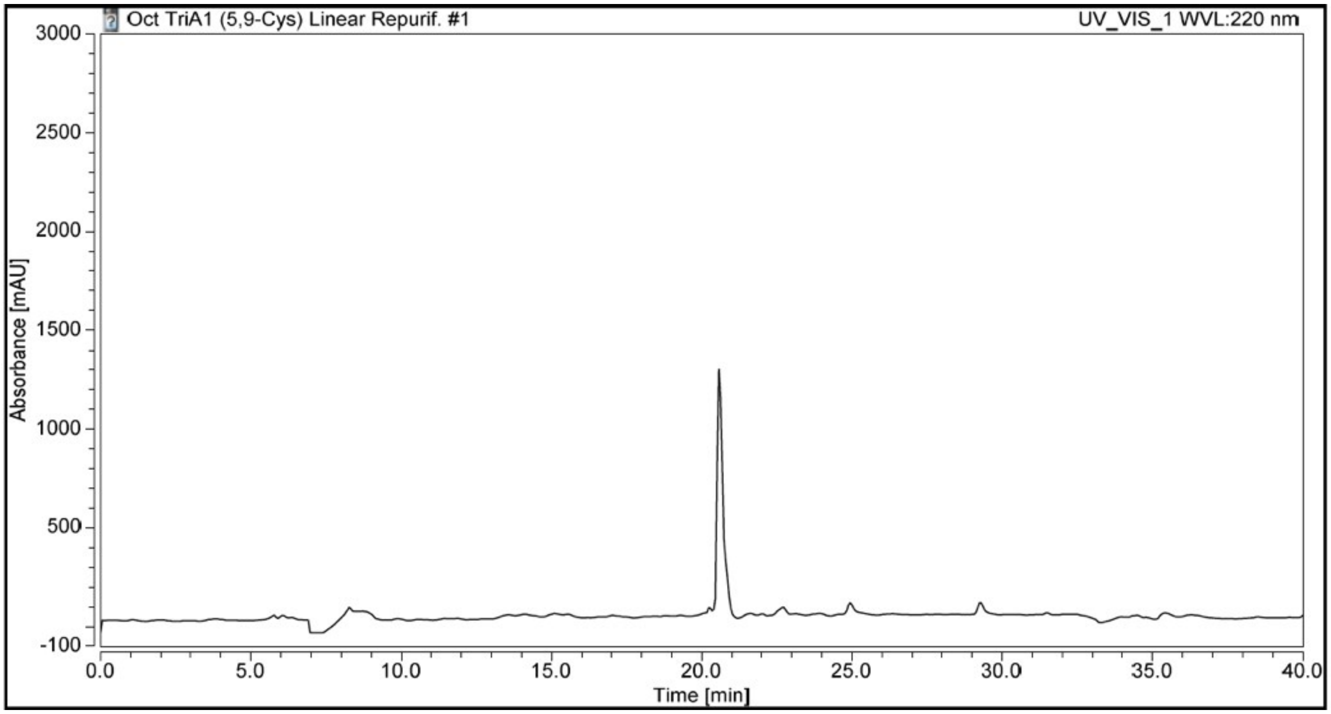

Fig. S5. HPLC trace showing the reinjection of purified Oct-TriA$_1$ (5-D-Cys, 9-Cys) (**4**). The peptide eluted as a single peak at 20.6 min using the HPLC method outlined in part III.

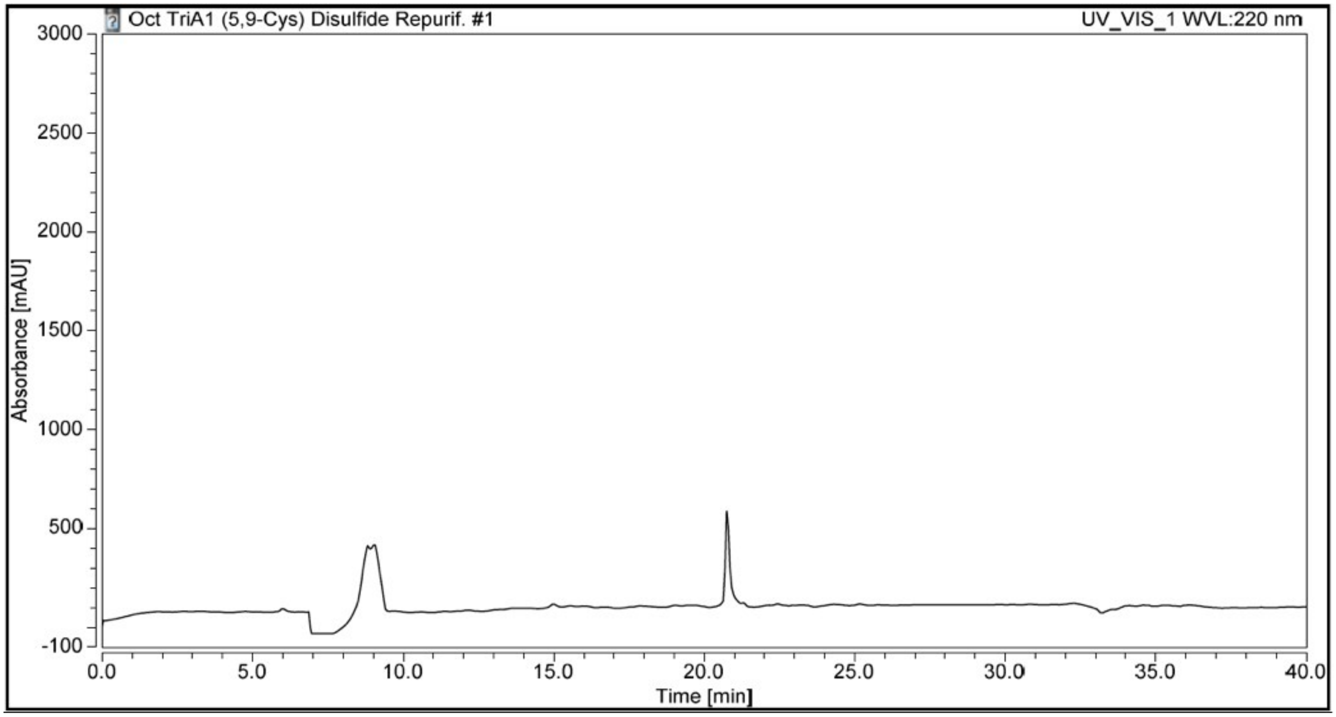

Fig. S6. HPLC trace showing the reinjection of purified Oct-cTriA$_1$ (SS) (**5**). The peptide eluted as a single peak at 20.8 min using the HPLC method outlined in part III.

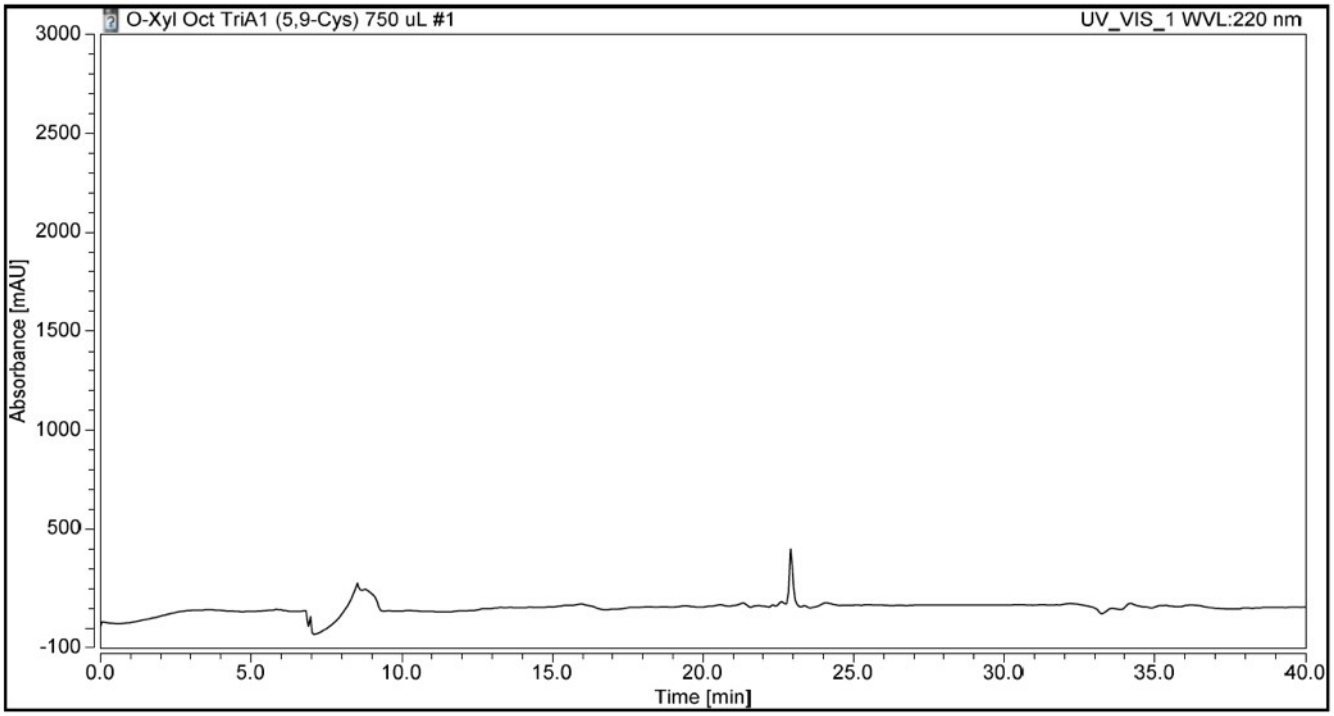

Fig. S7. HPLC trace showing the reinjection of purified of Oct-cTriA$_1$ (*o*-Xyl) (**6**). The peptide eluted as a single peak at 22.9 min using the HPLC method outlined in part III.

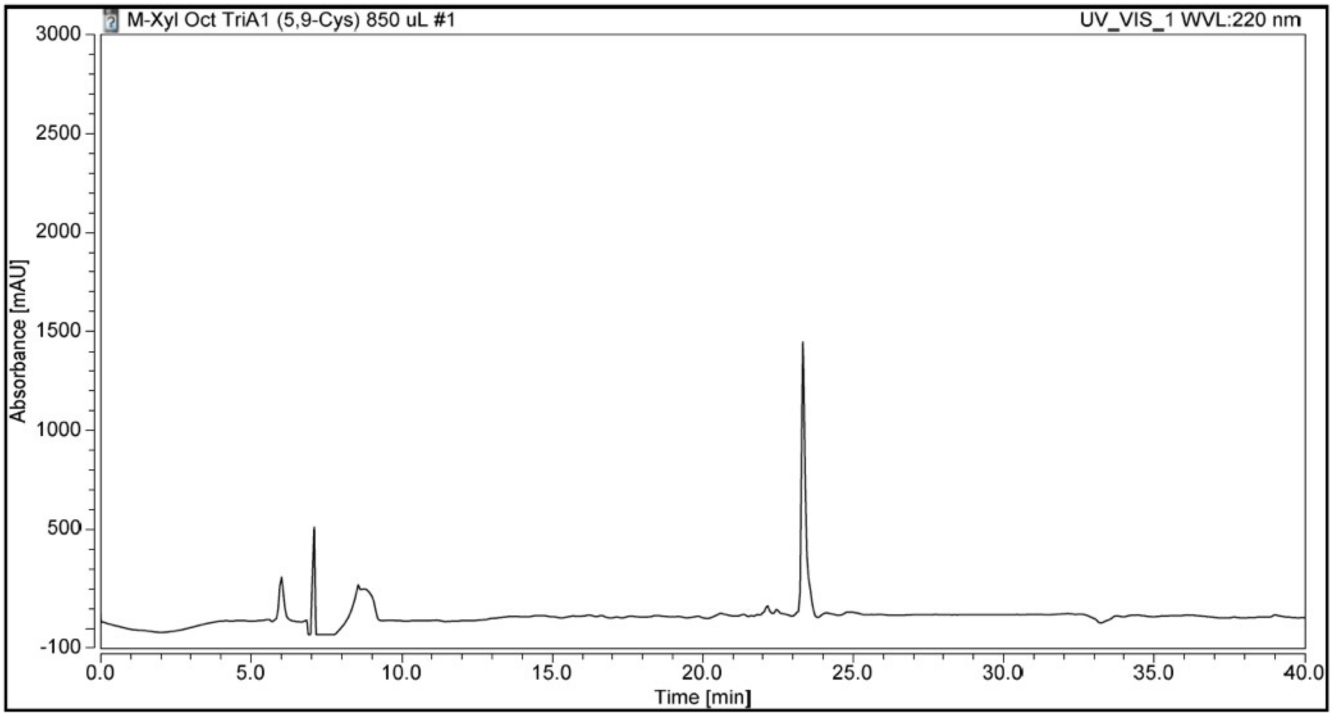

Fig. S8. HPLC trace showing the reinjection of purified Oct-cTriA$_1$ (*m*-Xyl) (**7**). The peptide eluted as a single peak at 23.3 min using the HPLC method outlined in part III.

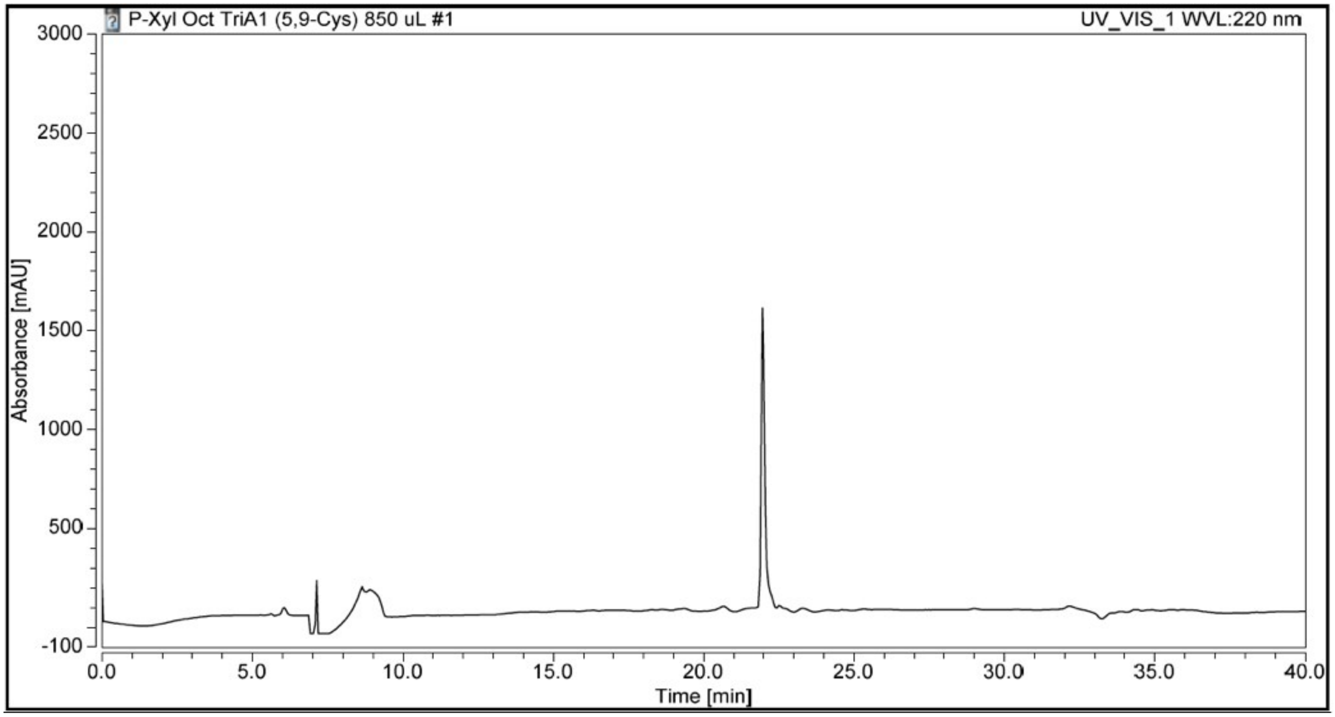

Fig. S9. HPLC trace showing the reinjection of purified Oct-cTriA$_1$ (*p*-Xyl) (**8**). The peptide eluted as a single peak at 22.0 min using the HPLC method outlined in part III.

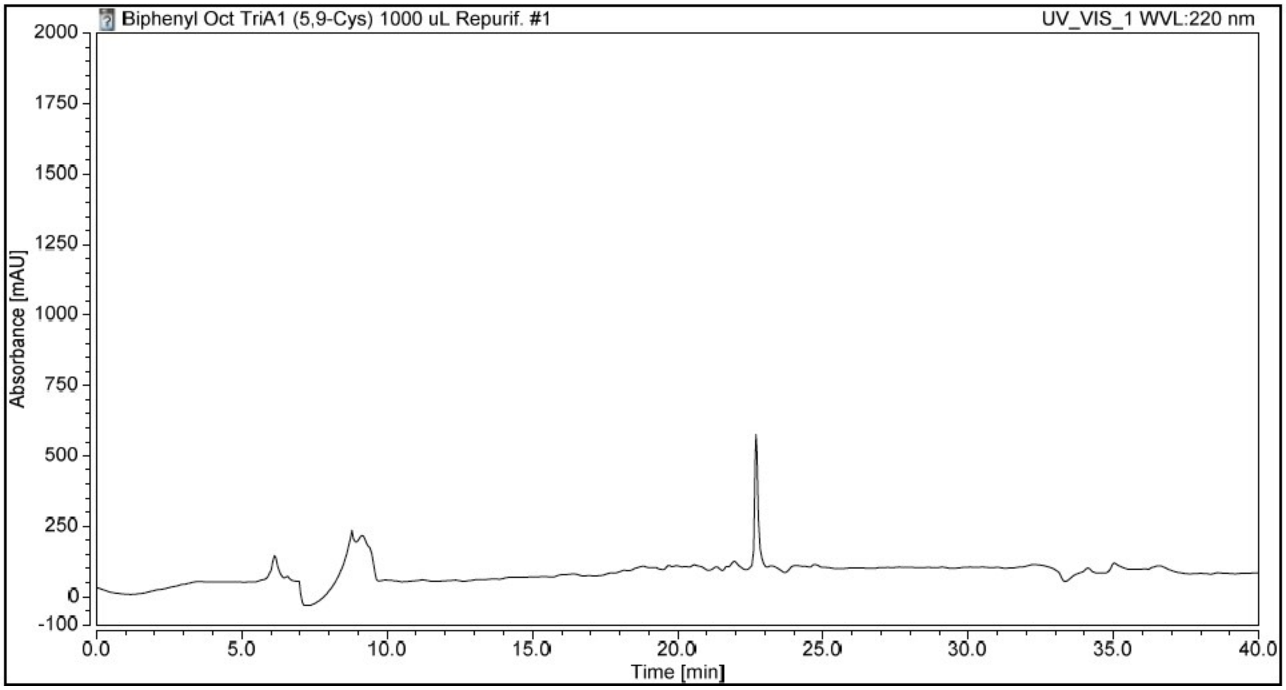

Fig. S10. HPLC trace showing the reinjection of purified Oct-cTriA$_1$ (biphenyl) (**9**). The peptide eluted as a single peak at 22.7 min using the HPLC method outlined in part III.

References:

1. N. Goudreau, C. Brochu, D. R. Cameron, J. S. Duceppe, A. M. Faucher, J. M. Ferland, C. Grand-Maître, M. Poirier, B. Simoneau and Y. S. Tsantrizos, *J Org Chem,* 2004*,* **69**, 6185-6201.
2. S. A. Cochrane, Z. Huang and J. C. Vederas, *Org Biomol Chem,* 2013*,* **11**, 630-639.
3. Y. X. Li, Z. Zhong, P. Hou, W. P. Zhang and P. Y. Qian, *Nat Chem Biol,* 2018*,* **14**, 381-387.
4. D. J. Derksen, J. L. Stymiest and J. C. Vederas, *J Am Chem Soc,* 2006*,* **128**, 14252-14253.
5. I. Wiegand, K. Hilpert and R. E. Hancock, *Nat Protoc,* 2008*,* **3**, 163-175.
